# Tetanus and Tetanic Spasms

**Published:** 1865-01

**Authors:** M. H. Houston

**Affiliations:** P. A. C. S.


### CONFEDERATE STATES MEDICAL & SURGICAL JOURNAL.

VOL. II. RICHMOND, JANUARY, 1865. No. 1.

### ORIGINAL COMMUNICATIONS.

ART. I.—*Tetanus and Tetanic Spasms.* By M. H. HOUSTON, P. A. C. S.

True Tetanus manifests itself by tonic contractions of the voluntary muscles, believed to be dependent on some persistent organic change in the medulla oblongata and spinal column. The essential character of this change has eluded thus far the researches of the scalpel and microscope, whilst its removal has baffled, in most cases, the best directed efforts of the most skillful physicians. It may be inflammation, it may be malassimilation, or it may be imperfect transformation of the tissues. Whatever it may be, it seems to have its seat in the nerve centres, and not in the branches. As indicative of its central seat, and in acknowledgment of his ignorance of its true nature, Mr. Travers, more than thirty years ago, gave it the name of constitutional irritation, and, for any light that has since been thrown upon the subject, the name may as well be retained.

This centric or constitutional irritation may be idiopathic, requiring for its development some accidental cause; or it may be traumatic, in which case, the central lesion is the result of long, continued irritation of the peripheral nerves.—Hence, all cases following wounds, are not to be considered traumatic, as in many cases the wound only acts as the exciting cause. The mine having been laid by some remote cause, the injury acts as a spark in producing the explosion.

A very concise history of cases will best illustrate the difference between these two varieties of tetanus. For reasons which need not be mentioned, I am compelled to give them entirely from memory.

*Case* 1.—A stout coal digger, between thirty and forty years of age, and apparently of good constitution, had compound fracture of the left leg, from the falling in of a coal bank. He had some other slight injuries, but the pain which he suffered during the first two days, was not greater than is usually consequent on such accidents. On the third day, he complained of sore throat and a sense of stiffness about the muscles of the jaw, with some difficulty of swallowing. Tetanus was gradually but steadily developed, and in three or four days it proved fatal.

*Case* 2.—A healthy looking negro girl, sixteen years of age, ran a nail into the bottom of her foot. A piece of fat bacon was applied to the wound and she was permitted to go about as usual. On the third day, she complained of some difficulty of swallowing and *soreness all over.* The tetanic spasms were developed slowly and insidiously, and it was not until the fifth day that they could be considered well pronounced. The tonic spasms were more persistent and the paroxysmal less violent, than in other cases I had witnessed. She died ten days after the receipt of the injury.

*Case* 3.—A stage driver was thrown from the top of his stage, on a pile of stones, and had comminuted compound fracture of the right leg. There were many sharp spiculæ of bone, with considerable laceration of the soft parts. He suffered great pain in the seat of injury, and for some days there was much twitching of the muscles of the limb. This painful condition of things continued, with varying severity, until nearly the end of the fourth week, when tetanus set in, with its ordinary symptoms, and put an end to his life in two or three days.

This is a concise statement of the important features in these three cases, the two first of which I consider as idiopathic, and the last purely traumatic. In the first, the centric disease—the constitutional irritation of Mr. Travers—existed at the time the injury was received, and the wound acted as the exciting cause only; in the last, the centric disease was produced by the protracted irritation extending from the periphery to the centre, and the injury was both the remote and the exciting cause. In the two first cases, the spasms occurred too soon after the receipt of the injury, and the local irritation was too slight to justify the belief that the centric disease was caused by the local injury.

In the third case, the local irritation was greater than in the other two, and yet nearly five weeks elapsed before any manifestations of centric disease displayed themselves.

This distinction is important as bearing upon the question of amputation, in such cases as involve the certainty of protracted, painful, local irritation. The history of tetanus proves that amputation would have had no effect in preventing the fatal result in the two first cases—indeed it seems more than probable, that any operation, would in itself have proved sufficient to bring into activity the latent constitutional irritation. Nor would amputation have prevented death in the last case, if performed after the development of the tetanic spasms, for the reason that the centric disease being established, its effects would continue after the removal of the traumatic cause. On the other hand, it is quite reasonable to believe, that if amputation had been performed at any time within the first three weeks after the accident, the constitutional irritation would have been avoided, and the developemnt of tetanus prevented. In determining, therefore, upon the propriety of operating in any particular case, the probability of secondary tetanus from protracted local irritation, presents an important consideration. If the term traumatic be retained, in all cases following wounds, then it may be laid down as a general rule, that any operation will prove worse than useless in primary development at whatever stage it be performed, whilst if early performed, it may prove efficient in arresting secondary tetanus.

But it may be asked, what evidence have we that in such cases as those just narrated, there exists any organic change in the nerve centres? In the absence of any appreciable anatomical lesion, we are left to infer its existence from a close observance of the symptoms, in their difference from other varieties of tonic contraction of the voluntary muscles.

Still, assuming that mere tonic contraction of the voluntary muscles, whether dependent or not upon organic change in the nerve centres, constitutes tetanus, we have other varieties of the disease which may as well be illustrated by the history of a few cases In this category I do not include those spasmodic contractions of the muscles of a limb, which so often follow fractures, and which are sometimes dignified with the name of tetanic spasms. These are just as independent of the condition of the nerve centres as are the cramps of a muscle from fatigue and exposure, to cold. They are, however, significant of the severity of local irritation, and should always be well considered in determining questions of amputation. They were well marked in the third case related, and yet tetanus did not supervene until near the end of the fourth week. In the class of cases to which I now refer, most of the voluntary muscles are involved, producing regular trismus and opisthotonos, of longer or shorter duration. The following case will be recognized by most practitioners as an old acquaintance:

*Case* 4.—Many years ago I was asked by a gentleman of no inconsiderable experience in his profession, to visit with him what he considered to be a case of tetanus. The patient was a strong looking girl, eighteen or nineteen years of age. At the time we entered her room, she presented an exaggerated appearance of both trismus and opisthotonos. The muscular contractions continued firm during many minutes, when they suddenly relaxed, and she fell into a state of perfect tranquillity. She continued thus quiescent for ten or fifteen minutes, when giving a sudden start, she immediately repeated the muscular phenomena above described. A few inquiries satisfied me that this was clearly a case of hysterical tetanus, dependent on dysmennorrhœa. The highly impressible nerve centres were aroused into activity by the uterine irritation, and diffused their influence through the voluntary muscles. As long as the uterine irritation lasted, the tonic contraction of the muscles continued with great violence. When one intermitted, the other followed its example, and both were renewed simultaneously. They were both permanently relieved by an injection of morphia in camphor emulsion.

*Case* 5.—A stout young Scotchman, twenty years of age, had a slight chill, which was followed by high fever, with intense pain in the head and back. On the second day I found him with violent tonic spasms, which had come on rather suddenly during the night. Both trismus and opisthotonos were complete, and somewhat exaggerated. On the third day a copious eruption of small-pox began to show itself, and in three days more, when the skin disease was fully developed, the tetanic spasms began to subside, and disappeared almost as rapidly as they had commenced. The tonic contractions continued without any intermission nearly four days. The patient passed through the regular stages of confluent small-pox without any return of the spasms, and had a good recovery.

In this unique case the tetanic spasms would seem to have been caused by the direct impression of an animal poison on the nerve centres. As soon as the central organs were relieved by the eruption of the poison on the skin, the spasms ceased, thus showing that no permanent change had been produced on the nerve centres.

*Case* 6.—Within the last few months I was called to see a negro woman, who was said to be *in a fit*. Being only a few steps distant, I went immediately, and found the woman in a chair in the yard, with all the voluntary muscles in a state of violent tonic contraction. At first I supposed it to be hysteria, but upon feeling no pulse at the wrist, and no sound of the heart, with my ear applied to the chest, I concluded there must be something more serious. In a few minutes the spasms relaxed entirely, the pulse returned to the wrist, the respiration became regular, and the patient was able to answer some questions. All that could be ascertained at the time was, that she had taken, what was supposed to be a dose of salts, a short time previously, and had eaten pretty freely of watermelon afterwards. Whilst in a state of relaxation, she was taken into the house and laid on a bed. After a few minutes the spasms returned with increased violence, respiration was again suspended, the pulse could not be felt at the wrist, the sounds of the heart could no longer be heard, and the patient continued in this state until death put an end to her sufferings. From first to last, the scene could not have lasted more than twenty minutes. Subsequent investigation proved that strychnine had been taken by mistake for salts. What time elapsed between the swallowing of the poison and the inception of the spasms could not be ascertained satisfactorily; so large a dose of the poison was longer in producing its specific effect than might have been reasonably anticipated. As far as I could judge, death was caused by tonic contraction of the respiratory muscles and the muscles of the heart.

It will be observed in this case, that notwithstanding the large dose of the poison, and the speedy manner in which it caused death, there was yet, during several minutes, an entire intermission in the tonic contraction of the muscles.

In the absence of any demonstrable anatomical lesions, all theories on the subject of tetanus must be, necessarily, more or less speculative. Notwithstanding this, we are not to relax in our exertions, or to become careless in our observations. Circumstantial evidence is sometimes more conclusive than positive testimony; and, we think, that in tetanus a careful study of the symptoms will enable us to determine, in most cases, the essential from the non-essential causes of the disease with as much certainty as we can do in most other affections. The difficulty of determining between functional and organic diseases of the heart, during life, is generally recognized, and yet a careful observance of all the symptoms will generally enable us to do so with much certainty.

The terms tetanus, tetanic convulsions and tetanic spasms, are used rather indiscriminately to designate every variety of tonic contraction of the voluntary muscles. With the view of procuring more definite ideas on the subject, I suggest the propriety of dividing the disease into the *organic* or *true tetanus*, and the *functional, sympathetic* or *simulated tetanus*—or, if it be preferred, the term *tetanic spasm* may be applied to the latter variety.

The *organic* or true tetanus may be subdivided into the *idiopathic* and traumatic, and the latter again into the primary and secondary traumatic. The functional, or simulated tetanus, will admit of as many varieties as there are causes to produce it; thus we have toxic tetanus, hysterical tetanus, &c., &c.

Under the head *organic* tetanus I would include all those cases, whether idiopathic or primary or secondary traumatic, in which the symptoms come on very gradually, and generally develope themselves with much regularity. Soreness of the throat, difficulty of deglutition, and sense of stiffness in the muscles, nearest the nerve centres, are first observed. These increase until tonic spasms are established, which gradually extend to the muscles of the trunk, and finally to those of the extremities. Although we have occasional paroxysms of increased muscular contraction, yet they are not so violent as in the functional disease, and are never followed by entire relaxation. There is no distinct intermission. When fatal, the spasms relax only in death; when not fatal, they relax in the same gradual manner in which they commenced, only the order of relaxation is reversed, and the muscles in the vicinity of the nerve centres are the last to yield. As a whole, the organic disease is less violent in its manifestations, although much more fatal in its results than the functional. In organic tetanus the spasms persist, notwithstanding the removal of the exciting cause; whilst in functional tetanus the removal of the exciting cause at once puts an end to the disease. In functional tetanus the spasms come on and go off more rapidly than in traumatic, while they are much more violent during their continuance. If there be intermission in the exciting cause, there is a corresponding intermission in the muscular contractions. The contractions commence with the exciting cause, and relax with its cessation. These constitute the principal differences in the muscular phenomena as they present themselves in these two divisions of tetanus, and I apprehend they are sufficient to enable us to distinguish the one from the other.

Perhaps it may be impossible to distinguish accurately between primary and secondary traumatic tetanus, and yet it will be safe to assume, as a general rule, that when the symptoms occur, within the first four or five days after an accident, the disease is primary, whilst if they occur after the fifth, or more certainly after the tenth day, it may be set down as secondary.

It was not my intention to say anything on the subject of treatment, but there are one or two suggestions growing out of the views just presented, which it may not be out of place to mention.

In functional tetanus, the object of the practitioner, of course, will be, to ascertain, and remove the exciting cause. This done, the disease will be cured. In organic tetanus, whether purely idiopathic or primary traumatic, local treatment can have no effect, except as it aids in relieving the constitutional irritation. In secondary traumatic tetanus amputation, if performed immediately after the appearance of the premonitory symptoms, and before the full establishment of the tonic spasms, may have the effect, by removing both the remote and the exciting cause, of preventing the full developement of the centric disease, and of assisting materially the effect of other remedies used for its removal.

The remedies upon which I should feel most disposed to rely would be, cupping over the medulla oblongata and spinal column, followed by a free use of the actual cautery at a white heat, with liberal doses of calomel and opium, and such other anodynes and anti-spasmodics as would be most likely to control the muscular contractions until the constitutional remedies could have time to produce their effect.

